# Quantitative Determination of Alkaloids in Lotus Flower (Flower Buds of *Nelumbo nucifera*) and Their Melanogenesis Inhibitory Activity

**DOI:** 10.3390/molecules21070930

**Published:** 2016-07-19

**Authors:** Toshio Morikawa, Niichiro Kitagawa, Genzoh Tanabe, Kiyofumi Ninomiya, Shuhei Okugawa, Chiaki Motai, Iyori Kamei, Masayuki Yoshikawa, I-Jung Lee, Osamu Muraoka

**Affiliations:** 1 Pharmaceutical Research and Technology Institute, Kindai University, 3-4-1 Kowakae, Higashi-osaka, Osaka 577-8502, Japan; morikawa@kindai.ac.jp (T.M.); kitagawa@koshiroseiyaku.co.jp (N.K.); ninomiya@phar.kindai.ac.jp (K.N.); okugawa_kameokanousan@koshiroseiyaku.co.jp (S.O.); motai@koshiroseiyaku.co.jp (C.M.); i.regretful.168.strike-swallow@docomo.ne.jp (I.K.); m-yoshikawa@leto.eonet.ne.jp (M.Y.); 2 Antiaging Center, Kindai University, 3-4-1 Kowakae, Higashi-osaka, Osaka 577-8502, Japan; 3 Koshiro Company Ltd., 2-5-8 Doshomachi, Chuo-ku, Osaka 541-0045, Japan; 4 Faculty of Pharmacy, Kindai University, 3-4-1 Kowakae, Higashi-osaka, Osaka 577-8502, Japan; g-tanabe@phar.kindai.ac.jp; 5 National Research Institute of Chinese Medicine, 155-1, Sec. 2, Linong St., Beitou District, Taipei 11221, Taiwan; lily@nricm.edu.tw

**Keywords:** lotus flower, *Nelumbo nucifera*, melanogenesis inhibitor, nuciferine, nornuciferine, quantitative analysis, carbamate salt

## Abstract

A quantitative analytical method for five aporphine alkaloids, nuciferine (**1**), nornuciferine (**2**), *N*-methylasimilobine (**3**), asimilobine (**4**), and pronuciferine (**5**), and five benzylisoquinoline alkaloids, armepavine (**6**), norarmepavine (**7**), *N*-methylcoclaurine (**8**), coclaurine (**9**), and norjuziphine (**10**), identified as the constituents responsible for the melanogenesis inhibitory activity of the extracts of lotus flowers (the flower buds of *Nelumbo nucifera*), has been developed using liquid chromatography-mass spectrometry. The optimum conditions for separation and detection of these 10 alkaloids were achieved on a *π*NAP column, a reversed-phase column with naphthylethyl group-bonded silica packing material, with CH_3_CN–0.2% aqueous acetic acid as the mobile phase and using mass spectrometry equipped with a positive-mode electrospray ionization source. According to the protocol established, distributions of these 10 alkaloids in the petal, receptacle, and stamen parts, which were separated from the whole flower, were examined. As expected, excellent correlations were observed between the total alkaloid content and melanogenesis inhibitory activity. Among the active alkaloids, nornuciferine (**2**) was found to give a carbamate salt (**2′′**) via formation of an unstable carbamic acid (**2′**) by absorption of carbon dioxide from the air.

## 1. Introduction

A Nymphaeaceae plant *Nelumbo nucifera* Gaertn. (common name “lotus” in English) is extensively cultivated in Eastern Asian countries [1,2,3]. All parts of this plant, including the leaves, stamens, flowers, seeds, and rhizomes, have been used as traditional medicines or vegetables for thousands of years [2,3,4]. The lotus flower, the flower buds of *N. nucifera*, has been used for the treatment of vomiting blood, bleeding caused by internal and external injuries, and various skin diseases, and also as a sedative and an anti-inflammatory agent in traditional Asian medicines [2]. In the course of our studies on the bioactive constituents from the flower buds of *N. nucifera*, we have isolated several alkaloids, e.g., nuciferine (**1**), nornuciferine (**2**), *N*-methylasimilobine (**3**), asimilobine (**4**), pronuciferine (**5**), and armepavine (**6**), with melanogenesis inhibitory activities in theophylline-stimulated murine B16 melanoma 4A5 cells [2]. As a result of the increasing interest in lotus flower as a possible cosmetic for skin whitening, there is a strong demand for efficient quality control measurements to ensure the authenticity and content of the active constituents in such products, and to verify the labeled claims. In this paper, we propose a simple, rapid, and precise analytical method for liquid chromatography-mass spectrometry (LC-MS) simultaneous quantitative determination of five aporphine alkaloids (**1**–**5**) and five benzylisoquinoline alkaloids, (**6**), norarmepavine (**7**), *N*-methylcoclaurine (**8**), coclaurine (**9**), and norjuziphine (**10**), using a one-step sample preparation procedure.

## 2. Results and Discussion

### 2.1. Isolation of Principal Alkaloids *(**1**–**10**)* from Lotus Flower

To obtain the principal alkaloids (**1**–**10**), an isolation procedure from this plant material was newly developed in this study by modifying the previously reported method [2]. Thus, dried flower buds of *N. nucifera* were extracted with methanol under reflux to obtain a methanol extract (9.22% from the dried material). The methanol extract was partitioned into a mixture of EtOAc and 3% aqueous tartaric acid (1:1, *v/v*) to furnish an acidic EtOAc-soluble fraction (2.88%) and an acidic aqueous solution. The pH of the aqueous solution was adjusted to 9 with saturated aqueous Na_2_CO_3_ and then extracted with CHCl_3_ to obtain a CHCl_3_-soluble fraction (0.97%). The aqueous layer was further extracted with *n*-BuOH to obtain an *n*-BuOH-soluble fraction (0.62%). As shown in Table 1, the methanol extract was found to inhibit theophylline-stimulated melanogenesis (IC_50_ = 5.6 µg/mL) without cytotoxicity (cell viability in the 3-(4,5-dimethylthiazol-2-yl)-2,5-diphenyltetrazolium bromide (MTT) assay: 103.3 ± 7.1%) at 100 µg/mL. Through bioassay-guided separation, the CHCl_3_-soluble fraction (IC_50_ = 0.37 µg/mL) was found to be more active than the EtOAc *n*-BuOH-soluble fractions (IC_50_ = 11.1 and 13.7 µg/mL, respectively).

**Figure 1:**
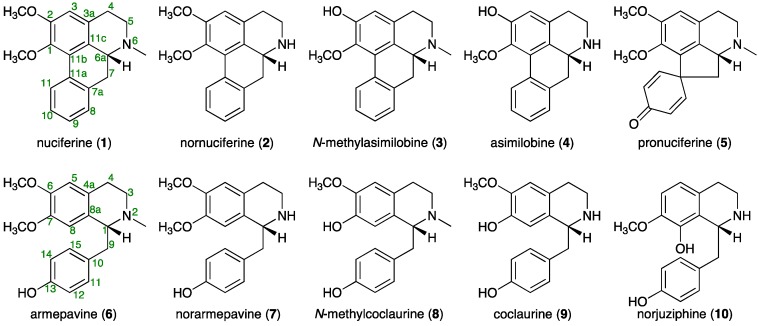
Aporphine and benzylisoquinoline alkaloids (**1**–**10**) from lotus flower.

The active CHCl_3_-soluble fraction was subjected to normal-phase silica gel, reversed-phase ODS column chromatography, and finally HPLC to furnish nuciferine (**1**, 0.1028%) [2,5,6], nornuciferine (**2**, 0.0821%) [2,5,6], *N*-methylasimilobine (**3**, 0.0094%) [2,7], asimilobine (**4**, 0.0345%) [2,6,8], pronuciferine (**5**, 0.0195%) [2,9], armepavine (**6**, 0.0170%) [2,10], norarmepavine (**7**, 0.0616%) [10,11], *N*-methylcoclaurine (**8**, 0.0053%) [12], coclaurine (**9**, 0.0042%) [10,13], and norjuziphine (**10**, 0003%) [14] (Figure 1).

### 2.2. Simultaneous Quantitative Analysis of 10 Alkaloids *(**1**–**10**)* in Lotus Flowers

**Figure 2:**
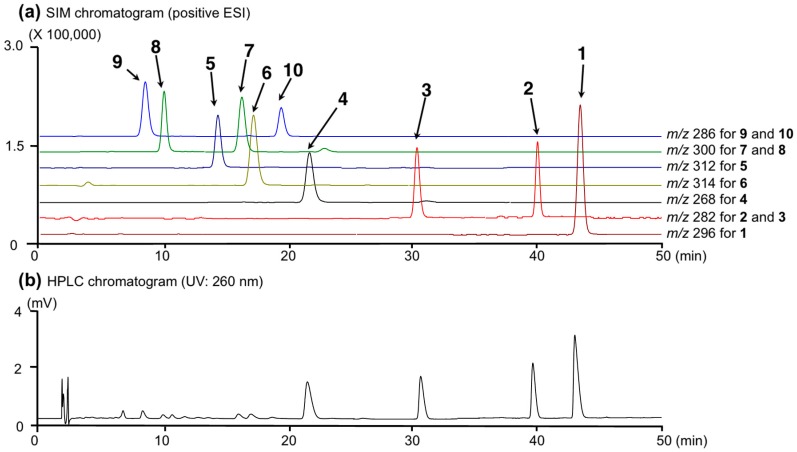
A typical LC-MS chromatogram of a standard solution mixture (each 10 µg/mL) of alkaloids (**1**–**10**). (**a**) SIM chromatogram (positive ESI); (**b**) HPLC chromatogram (UV: 260 nm).

To provide sufficient purity for quantitative analysis, the hydrochlorides of these alkaloids (**1**–**10**) were prepared by reported method [10]. As shown in Figure 2, typical LC-MS chromatograms for a standard solution mixture under UV (260 nm) and MS detections by electrospray ionization (ESI) MS under the positive mode demonstrated good baseline separation for all peaks. Each peak was observed at the following retention time and quasimolecular ion peak ([M + H]^+^) (*t*_R_: **1** (43.1 min, *m/z* 296), **2** (39.5 min, *m/z* 282), **3** (29.7 min, *m/z* 282), **4** (21.3 min, *m/z* 268), **5** (13.9 min, *m/z* 312), **6** (16.9 min, *m/z* 314), **7** (15.9 min, *m/z* 300), **8** (9.9 min, *m/z* 300), **9** (8.3 min, *m/z* 286), and **10** (18.8 min, *m/z* 286)). These peaks were unambiguously assigned by comparison of their retention times with those of authentic specimens [2].

Prior to analysis, extraction conditions were examined to optimize the extracts′ quality in association with the contents of the alkaloids (**1**–**10**). The extraction efficacies were compared for three solvent systems (methanol, 50% aqueous methanol, and water) under two different conditions (reflux for 120 min or sonication for 30 min, each twice). As shown in Table 2, “reflux in methanol” afforded the highest contents of the active alkaloids (**1**–**10**). Therefore, all the analytical samples were prepared by employing the method “reflux in methanol for 120 min”.

Some analytical parameters, such as linearity and limit of quantitation of the developed method, were evaluated as shown in Table 3. The calibration curve was linear in the range studied (0.5–50 µg/mL) showing a correlation coefficient (*R*^2^) of greater than 0.9996 for each constituent. Linear regression equations of their calibration curves for each constituent are described in Table 3, where *y* is the peak area and x is the concentration of the analyte. The detection and quantitation limits were estimated to be 0.17–0.90 and 0.51–2.65 ng, respectively, indicating sufficient sensitivity of this method. The relative standard deviation (RSD) values were 0.25%–1.36% for intra-day and 0.39%–1.40% for inter-day assays. Accuracy was determined in recovery experiments using the methanol extract of NN-1. As shown in Table 4, recovery rates of 92.3%–105.8% were obtained, with RSD values of lower than 1.6%.

According to the protocol thus established, contents of the alkaloids (**1**–**10**) collected in two different regions (NN-1 in Thailand; NN-5 in Taiwan) were measured. The assay was found to be reproducible, precise, and readily applicable to the quality evaluation of lotus flower′s extracts. As shown in Table 5, *N*-methylcoclaurine (**8**, NN-1: 5.73 mg/g in dry material; NN-5: 2.88 mg/g) was the richest constituent among the alkaloids (**1**–**10**). The total alkaloid content in the Thai (NN-1: 14.96 mg/g) and Taiwanese (NN-5: 3.53 mg/g) samples were quite different. However, a more extensive study would be required to confirm that this result was due to differences between regions. To characterize the distribution of the alkaloids (**1**–**10**) in the flower, the whole flower parts (NN-1 and NN-5) were separated into petals (NN-2 and NN-6), receptacles (NN-3 and NN-7), and stamens (NN-4 and NN-8); then, quantitative analysis of each separated sample was performed. It was found that the alkaloids (**1**–**10**) were mainly contained in the petal part. Furthermore, other parts of the lotus plant (e.g., leaf (NN-9), fruit (NN-10 and 11), and embryo parts (NN-12), which are used for traditional medicines) were also examined. It was found that the total alkaloid content of the leaf (NN-9: 1.20 mg/g), fruit (NN-10 and NN-11: each less than the quantitation limit), and embryo parts (NN-12: 0.64 mg/g) of *N. nucifera* were lower than those of the flower buds (NN-1 and NN-5) (Table S1).

### 2.3. Ammonium Carbamate Salt *(**2′′**)* Formation from the Free Alkaloid *(**2**)*

**Figure 3:**
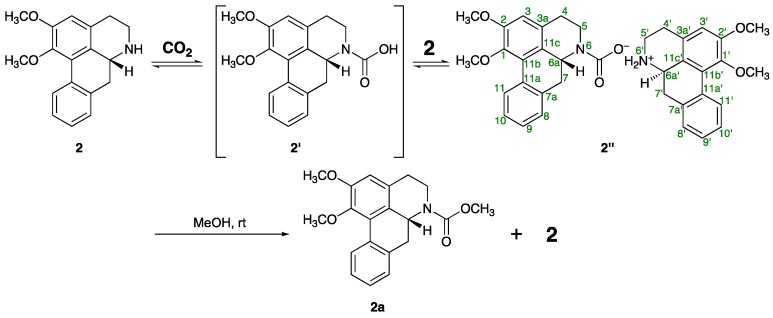
Chemical transformation of nornuciferine (**2**) into its ammonium carbamate salt (**2′′**) and to methyl carbamate (**2a**).

The gradual transformation of one of the alkaloids isolated in this study, nornuciferine (**2**), into a highly polar material **2′′** was observed, when **2** was exposed to the atmosphere in deuterated chloroform (CDCl_3_) at room temperature. After three weeks of standing in CDCl_3_, compound **2′′** was obtained as a main product (Figure 3). As summarized in Table 6, ^1^H- and ^13^C-NMR spectra of **2′′** suggested that there are two kinds of parts derived from the nornuciferine framework in the structure of **2′′**. Thus, with respect to one of the nornuciferine parts, five carbons α and β to the nitrogen atom appeared as pair signals [δ_C_: 29.9/30.2 (C-4), 41.8/44.4 (C-5), 54.9/55.8 (C-6a), 33.9/35.9 (C-7), 124.8/124.9 (C-11c)] in the ^13^C-NMR spectrum of **2′**. Additionally, a pair of signals, which corresponded to an amide type carbonyl carbon, was also observed at δ_C_ 157.5 and 160.1. The chemical shift of the signals suggested that the nitrogen atom of **2** was functionalized as a carbamate anion by the CO_2_ uptake from the atmosphere. On the other hand, in the ^1^H-NMR spectrum of **2′′** a downfield shift owing to the ammonium ion formation was observed with respect to the signals due to C-5′ methylene (at δ_H_ 3.24 and 3.87) and C-6a′ methine (at δ_H_ 4.29) protons of the other nornuciferine parts as compared with those of **2** [δ_H_: 3.01 and 3.40 (H_2_-5), 3.85 (H-6a)]. Two broad singlets, which appeared at the highly-deshielded regions (δ_H_ 9.96 and 10.84), were due to acidic protons, which also support the ammonium ion structure depicted in Figure 3. The anticipated structure of **2′** was strongly supported by the IR spectrum, which showed N^+^–H and C=O stretching absorptions at 2720–2500 and 1721 cm^−1^, respectively. Moreover, the positive ion part of **2′′** was detected as a Na adduct ion signal at *m/z* 282.1483 [M − C_19_H_18_NO_4_]^+^ (calcd for C_18_H_20_O_2_, 282.1489) in the positive ESI mode; however, a signal due to the carbamate group was not detected in the negative ESI mode. Fortunately, the negative ion part of **2′′** could be confirmed as the corresponding methyl carbamate **2a**. Thus, **2′′** easily gave a 1:1 mixture of methyl carbamate **2a** and original amine **2** by treatment with methanol at room temperature (Figure S1). As shown in Table 6, compound **2a** showed similar ^13^C-NMR spectroscopic properties to those of **2′′** and/or **2**, except for a singlet (δ_C_ 52.6) due to the methyl carbon of the NCO_2_*C*H_3_ moiety, which was confirmed by the correlation between the singlet at δ_H_ 3.76 due to the methyl protons and a singlet at δ_C_ 156.0 due to carbonyl carbon in the HMBC of **2a**. In the positive ESIMS of **2a**, a quasimolecular ion peak was observed at *m/z* 362.1361 [M + Na]^+^ (calced for C_20_H_21_NO_4_Na, 362.1363).

**Figure 4:**
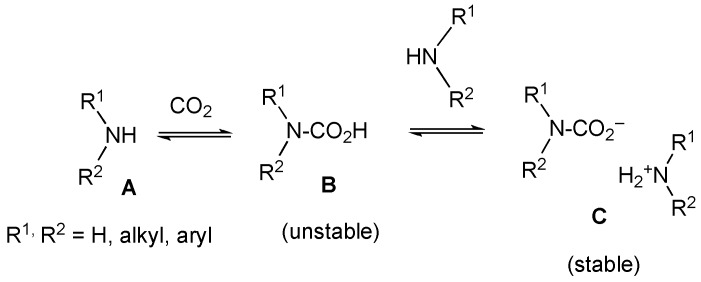
The reported reactivity of amines with CO_2_.

It is well known that ammonia, primary amines, or secondary amines (**A**) absorb CO_2_ to transform into the corresponding carbamic acids (**B**), which easily react with the original amines to produce stable carbamic acid ammonium salts and (**C**) as shown in Figure 4 [15,16,17,18,19,20,21,22,23,24]. Therefore, it is reasonable to anticipate that the product **2′′** forms via an acid-base reaction between original amine **2** and an unstable carbamic acid (**2′**), which was obtained by the CO_2_ absorption reaction with the nitrogen atom of **2**.

### 2.4. Effects of the Hydrochlorides of These Alkaloids *(**1**–***10***)* and ***2a*** on Theophylline-Stimulated Melanogenesis Inhibitory Activity

To clarify the efficacy of the established quantitative analysis of 10 alkaloids (**1**–**10**) as a quality control for lotus flower, correlations between the total alkaloid contents and the melanogenesis inhibitory activities of the corresponding extracts were examined. Previously, we have reported the melanogenesis inhibitory activities of free nuciferine (**1**, IC_50_ = 15.8 µM), nornuciferine (**2**, 62.9 µM), *N*-methylasimilobine (**3**, 14.5 µM), asimilobine (**4**, >100 µM), pronuciferine (**5**, 47.9 µM), and armepavine (**6**, 25.6 µM) [2]. Since the hydrochlorides of these alkaloids (**1**–**10**), which were of higher purity and stability than those of the corresponding free alkaloids, were used for the standard samples of the present quantitative analysis, the melanogenesis inhibitory activities of these hydrochloride salts were newly examined. It was found that aporphine alkaloids, nuciferine (**1**, IC_50_ = 7.1 µM) and nornuciferine (**2**, 3.9 µM), and benzylisoquinoline alkaloids, armepavine (**6**, 6.5 µM), norarmepavine (**7**, 7.5 µM), *N*-methylcoclaurine (**8**, 6.5 µM), and coclaurine (**9**, 3.9 µM), were found to show relatively strong inhibitory activities without notable cytotoxic effects at the effective concentration (Table 7). These alkaloids were more potent than arbutin (IC_50_ = 174 µM), a commercially used melanogenesis inhibitor used as a positive control [25,26,27]. Among them, **2** and **9** showed especially strong activity, which showed more than 40 times greater than that of arubutin. Previously, the naturally-occurring aporphine alkaloid, 4,5-didehydroguadiscine (IC_50_ = 4.7 µM) isolated from *Hornschuchia oblique*, has been reported [28]. To the best of our knowledge, compounds **2** and **9** are the most potent melanogenesis inhibitors within this class of natural products.

### 2.5. Effects on Mushroom Tyrosinase

To characterize the mode of action of melanogenesis inhibitory activities of the alkaloids, inhibitory effects on (i) enzymatic tyrosinase activity and (ii) expressions of tyrosinase-related proteins (TRPs) e.g. tyrosinase, TRP-1, and TRP-2 were examined.

A copper-containing enzyme tyrosinase is a key enzyme in melanin biosynthesis involved in determining the color of skin and hair. It catalyzes oxidation of both l-tyrosine and l-DOPA, following another oxidation of l-DOPA to dopaquinone and, finally, oxidative polymerization via several dopaquinone derivatives to yield melanin. Tyrosinase inhibitors are being clinically used for the treatment of several dermatological disorders associated with melanin hyperpigmentation. The tyrosinase inhibitor kojic acid is commonly used as an additive in cosmetics for skin whitening and/or depigmentation [25,26,27]. As shown in Table 8, none of the alkaloids showed inhibitory activities when using both l-tyrosine and l-DOPA as substrates. This suggests that tyrosinase inhibition is barely involved in the mechanisms of action of these melanogenesis inhibitors.

### 2.6. Effects on Expression of Tyrosinase, TRP-1, and TRP-2

The TRP enzyme family (tyrosinase, TRP-1, and TRP-2) catalyze the major steps in melanin synthesis [25,26,27]. To clarify the mechanisms of action of these active constituents, we examined the effects of the principal alkaloids (**1**, **2**, **6**, **7**, and **9**) on expression of mRNAs for tyrosinase, TRP-1, and TRP-2 in B16 melanoma 4A5 cells. Except for compound **7**, the suppression tendency of mRNA expression for tyrosinase in these alkaloids was observed as presented in Table 9.

### 2.7. Correlation between the Melanogenesis Inhibitory Activity and Total Contents of Alkaloids *(**1**–**10**)* in Lotus Flower Extracts

**Figure 5:**
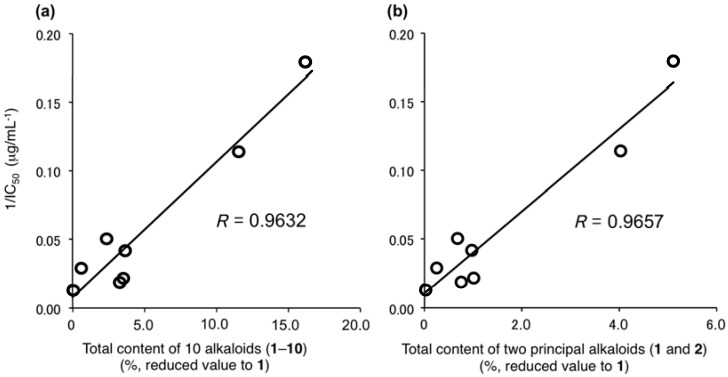
Correlations between the melanogenesis inhibitory activities and total content. (**a**) Total content (%) of 10 alkaloids (**1**–**10**); (**b**) Total content (%) of two principal alkaloids (**1** and **2**). Total contents (%) of the alkaloids are presented in values reduced to the content of nuciferine (**1**), calculated based on the ratio of IC_50_ values (µg/mL) against melanogenesis inhibitory activities.

The inhibitory effects of the methanol extracts of the lotus flowers (NN-1–8) on theophylline-stimulated melanogenesis were examined. As a result, the IC_50_ values were detected in all the lotus flower samples in the ranges of 5.8–78.9 µg/mL (Table 10). In Figure 5, correlations between the total content of 10 alkaloids (value reduced to **1**) and the melanogenesis inhibitory activities (1/IC_50_) of the corresponding extracts were plotted. As expected, excellent correlations were observed between the total content and the inhibitory activities (*R* = 0.9632). As the minimum involvement, these correlations were shown between the content of two principal alkaloids (**1** and **2**) and the activities (*R* = 0.9657). In addition, the methanol extracts from the leaf and fruit parts of *N. nucifera* (NN-9–11) did not show melanogenesis inhibitory activities (IC_50_ > 100 µg/mL, Table S2). On the other hand, despite the fact that the methanol extract from the embryo of *N. nucifera* (NN-12) contained scarcely any alkaloids (**1**–**10**) (vide supra), potent melanogenesis inhibitory activity was observed (IC_50_ = 4.5 µg/mL, Table S2). This evidence suggested that other melanogenesis inhibitory active constituents are included in the embryo part.

## 3. Materials and Methods

### 3.1. General Experimental Procedures

The following instruments were used to obtain physical data: melting points, Yanagimoto micromelting point apparatus (Yanaco New Science Inc., Kyoto, Japan); specific rotations, SEPA-300 digital polarimeter (Horiba Ltd., Kyoto, Japan, *l* = 5 cm); UV spectra, UV-1600 spectrometer (Shimadzu Co., Kyoto, Japan); IR spectra, FTIR-8100 spectrometer (Shimadzu Co.); ESIMS and HRESIMS, Exactive Plus mass spectrometer (Thermo Fisher Scientific Inc., Waltham, MA, USA); ^1^H-NMR spectra, JNM-ECA800 (800 MHz), JNM-ECA500 (500 MHz), and JNM-ECS400 (400 MHz) spectrometers (JEOL Ltd., Tokyo, Japan); ^13^C-NMR spectra, JNM-ECA800 (200 MHz), JNM-ECA500 (125 MHz), and JNM-ECS400 (100 MHz) spectrometers (JEOL Ltd.) with tetramethylsilane as an internal standard; HPLC detector, SPD-10A*vp* UV-VIS detector (Shimadzu Co.); HPLC column, Cosmosil 5C_18_-MS-II (Nacalai Tesque, Inc., Kyoto, Japan), 4.6 mm × 250 mm i.d. and 20 mm × 250 mm i.d. for analytical and preparative studies, respectively.

The following experimental conditions were used for chromatography (CC): ordinary-phase silica gel column chromatography, silica gel 60N (Kanto Chemical Co., Tokyo, Japan; 63–210 mesh, spherical, neutral); reverse-phase silica gel CC, Chromatorex ODS DM1020T (Fuji Silysia Chemical, Aichi, Japan; 100–200 mesh); normal-phase TLC, pre-coated TLC plates with silica gel 60F_254_ (Merck, Darmstadt, Germany; 0.25 mm); reversed-phase TLC, pre-coated TLC plates with silica gel RP-18 F_254S_ (Merck, 0.25 mm); reversed-phase HPTLC, pre-coated TLC plates with silica gel RP-18 WF_254S_ (Merck, 0.25 mm), detection was carried out by spraying 1% Ce(SO_4_)_2_–10% aqueous H_2_SO_4_ on the plates, followed by heating.

### 3.2. Plant Materials

The flower buds of *Nelumbo nucifera* collected in Nakhon Ratchasima, Thailand, in 2011, and were abbreviated as followings: NN-1 (the whole flowers), NN-2 (the petals), NN-3 (the receptacles), and NN-4 (the stamens). The flower buds of *N. nucifera* collected in Taiwan, in 2011, and were as follows: NN-5 (the whole flowers), NN-6 (the petals), NN-7 (the receptacles), and NN-8 (the stamens). The other samples (NN-9–12) were purchased via Tochimoto Tenkaido Co., Ltd., Osaka, Japan, in 2013 and were abbreviated as follows: NN-9 [the leaves collected from Shandong province, China, in 2010 (Lot. No. 1111C129201)], NN-10 [the fruit collected from Hunan province, China, in 2012 (Lot. No. 1212C026701)], NN-11 [the fruit collected from Hunan province, China, in 2011 (Lot. No. 1206C026701)], and NN-12 [the embryos were collected from Jiangxi province, China, in 2010 (Lot. No. 1207128901)]. These plant materials were identified by one of the authors (M.Y.), and voucher specimens of them are on file in our laboratory. The materials were air-dried in a room under shade for more than a month.

### 3.3. Extraction and Isolation

Dried flower buds of *N. nucifera* (NN-1, 1.98 kg) were extracted four times with methanol (10 L) at room temperature for 24 h. Evaporation of the combined extracts under reduced pressure provided a methanol extract (182.75 g, 9.22%). An aliquot (168.51 g) of the methanol extract was partitioned into a mixture of EtOAc and 3% aqueous tartaric acid (1:1, *v/v*) to furnish an acidic EtOAc-soluble fraction (52.69 g, 2.88%) and an acidic aqueous solution. The aqueous solution was adjusted to pH 9 with saturated aqueous Na_2_CO_3_ and then extracted with CHCl_3_. Removal of the solvent in vacuo yielded a CHCl_3_-soluble fraction (17.80 g, 0.97%). The aqueous layer was extracted with *n*-BuOH, and removal of the solvent in vacuo yielded a *n*-BuOH-soluble fraction (12.29 g, 0.62). An aliquot (17.70 g) of the CHCl_3_-soluble fraction was subjected to normal-phase silica gel CC [450 g, CHCl_3_–28% NH_4_OH (500:1, *v/v*) → CHCl_3_–28% NH_4_OH (500:1, *v/v*)–MeOH (100:1 → 70:1 → 50:1 → 20:1 → 10:1 → 1:1, *v/v*) → MeOH] to give 13 fractions (Fr. 1 (441.6 mg), Fr. 2 [= nuciferine (**1**, 1571.1 mg, 0.1028%)], Fr. 3 (183.2 mg), Fr. 4 (318.3 mg), Fr. 5 (689.7 mg), Fr. 6 [= nornuciferine (**2**, 782.1 mg, 0.0512%)], Fr. 7 [= asimilobine (**4**, 508.8 mg, 0.0333%)], Fr. 8 (543.8 mg), Fr. 9 (983.3 mg), Fr. 10 [= norarmepavine (**7**, 559.2 mg, 0.0366%)], Fr. 11 (464.4 mg), Fr. 12 (3173.1 mg), and Fr. 13 (3212.7 mg)). The fraction 3 (183.2 mg) was purified by HPLC [UV (254 nm), MeOH–0.03% aqueous Et_2_NH (60:40, *v/v*)] to give **1** (83.6 mg, 0.0055%), **2** (15.2 mg, 0.0010%), and pronuciferine (**5**, 30.0 mg, 0.0020%). The fraction 4 (318.3 mg) was purified by HPLC [UV (254 nm), MeOH–0.03% aqueous Et_2_NH (40:60, *v/v*)] to give **1** (6.3 mg, 0.0004%) and *N*-methylasimilobine (**3**, 34.6 mg, 0.0024%). The fraction 5 (689.7 mg) was purified by HPLC [UV (254 nm), MeOH–0.03% aqueous Et_2_NH (40:60, *v/v*)] to give **2** (279.5 mg, 0.0262%), **3** (62.0 mg, 0.0067%), and **5** (181.3 mg, 0.0195%). The fraction 8 (453.8 mg) was purified by HPLC [UV (254 nm), MeOH–0.03% aqueous Et_2_NH (40:60, *v/v*)] to give armepavine (**6**, 260.3 mg, 0.0170%). An aliquot (599.6 mg) of the fraction 9 was purified by HPLC [UV (254 nm), MeOH–0.03% aqueous Et_2_NH (40:60, *v/v*)] to give **4** (19.2 mg, 0.0012%) and **7** (210.0 mg, 0.0137%). The fraction 11 (464.4 mg) was purified by HPLC [UV (254 nm), MeOH–0.03% aqueous Et_2_NH (40:60, *v/v*)] to give **7** (144.9 mg, 0.0113%), *N*-methylcoclaurine (**8**, 56.0 mg, 0.0044%), and norjuziphine (**10**, 10.1 mg, 0.0003%). An aliquot (512.3 mg) of the fraction 12 was purified by HPLC [UV (254 nm), MeOH–0.03% aqueous Et_2_NH (40:60, *v/v*)] to give coclaurine (**9**, 14.2 mg, 0.0058%) and **10** (4.5 mg, 0.0018%).

### 3.4. Preparation of Hydrochlorides of Alkaloids *(**1**–**10**)*

Preparation of the hydrochlorides as standard samples for quantitative analysis was carried out according to the procedure described previously but with a slight modification [10]. Free nuciferine (**1**, 29.5 mg, 0.1 mmol) was dissolved in a mixture of dichloromethane (1.0 mL) and ethanol (1.0 mL) at 0 °C. Concentrated HCl (150 µL) was added, and after stirring at 0 °C for 1 h, the reaction mixture was suction filtered with a Kiriyama funnel (Kiriyama glass Co., Tokyo, Japan), leaving a residue. The residue was washed with dichloromethane yielded a hydrochloride **1**, which was sufficiently pure for analysis. Through a similar procedure, hydrochlorides **2**–**10** were obtained from the corresponding free alkaloids (**2**–**10**). 

### 3.5. Standard Solution Preparation

Accurately weighed 2.00 mg of each hydrochloride salt of alkaloid (**1**–**10**) was introduced into a 20 mL volumetric flask, and the volume was made up with methanol; the solution being used as a stock standard solution (100 µg/mL). Aliquots of 50, 100, 500, 1000, and 5000 µL of the stock standard solution were transferred into 10 mL volumetric flasks and the volume was made up with methanol for use as working solutions (0.5, 1.0, 5.0, 10, and 50 µg/mL, respectively) for constructing calibration curves. For calibration, an aliquot of 2.0 µL of each solution was injected into the LC-MS system. Each peak was observed at following retention times: **1** (*t*_R_ 43.1 min), **2** (*t*_R_ 39.5 min), **3** (*t*_R_ 29.7 min), **4** (*t*_R_ 21.3 min) and **5** (*t*_R_ 13.9 min), **6** (*t*_R_ 16.9 min), **7** (*t*_R_ 15.9 min), **8** (*t*_R_ 9.9 min), **9** (*t*_R_ 8.3 min), and **10** (*t*_R_ 18.8 min).

### 3.6. Sample Preparation

An accurately weighed pulverized sample powders (ca. 2 g, conversion with loss on drying) was extracted with 20 mL of three solvent systems (methanol, 50% methanol, or water) under two different conditions (reflux for 120 min or sonication for 30 min, each twice), respectively. After centrifugation of the extracts at 3000 rpm for 5 min, the supernatants were combined and diluted to 100 mL with the extraction solvent. An aliquot (1 mL) of the extract solution was transferred into a 5 mL volumetric flask and made up to the volume with methanol. The solution was filtered through a syringe filter (0.45 µm), and an aliquot of 5.0 µL was subjected to the LC-MS analysis. The remaining extraction solution (90 mL) was evaporated in vacuo to calculate the extraction yields.

### 3.7. LC-MS Instruments and Conditions

An LC-20A series Prominence HPLC system (Shimadzu Co.) was equipped with a binary pump, a degasser, an autosampler, a thermostated column compartment, a UV detector, and a control module connected with a LCMS-2010EV mass spectrometer (Shimadzu Co.) equipped with an ESI interface. The chromatographic separation was performed on a Cosmosil *π*NAP column (5 µm particle size, 2.0 mm i.d. × 150 mm, Nakalai Tesque Inc.) operated at 40 °C with mobile phase A (acetonitrile) and B (H_2_O containing 0.2% acetic acid). The gradient program was as follows: 0 min (A:B 15:85, *v/v*) → 20 min (18:82, *v/v*) → 50 min (50:50, *v/v*). The flow rate was 0.2 mL/min and the injection volume was 2.0 µL. The detections were performed at 260 nm (UV) and under selected ion monitoring (SIM) by a positive-mode ESI-MS. The operating parameters for MS detection were as follows; nebulizing gas flow: 1.5 L/min, drying gas pressure: 0.15 MPa, CDL temperature: 250 °C, block heater temperature: 250 °C, interface voltage: −3.5 kV, CDL voltage: constant-mode, Q-array DS and RF voltage: Scan-mode. 

### 3.8. Calibration and Validation

The standard curves were prepared over concentration ranges of 0.5–50 µg/mL with five different concentration levels. Standard curves were made on each analysis day. Linearity for each compound was plotted using linear regression of the peak area versus concentration. The coefficient of correlation (*R*^2^) was used to judge the linearity. The detection limit and quantitation limit for each analyte were determined by the signal-to-noise (*S/N*) ratio for each compound by analyzing a series of diluted standard solutions until the *S/N* ratios were about 3 and 10, respectively, based on a 2 µL injection. Precision and accuracy of the analytical method were tested using a homogeneous extract of NN-1. The intra- and inter-day precisions were determined by estimating the corresponding responses five times on the same day and on five different days, respectively (Table 3). The recovery rates were determined by adding analytes of three different concentrations (10, 15, and 20 µg/mL) to the sample solution (Table 4).

### 3.9. Reaction of Free Alkaloid *(**2**)* with CO_2_ in an Air Atmosphere

A free alkaloid **2** (50.0 mg, 0.718 mmol) was dissolved in CDCl_3_ (5.0 mL) under an air atmosphere; the resulting solution was kept at room temperature. The reaction was monitored by HPLC analysis: column (Cosmosil 5C_18_ MS-II (4.6 mm × 250 mm, i.d., Nakalai Tesque Inc.)); detection (UV (254 nm)); mobile phase (MeOH–0.03% aqueous Et_2_NH (70:30, *v/v*)); column temperature (room temperature); and flow rate: (1.0 mL/min). The monitoring results are shown in Figure S1.

After three weeks, the reaction mixture was condensed under reduced pressure to give a crude reddish brown solid (10.0 mg). The analytical sample of **2′′** (2.3 mg) was obtained as colorless needles by recrystallization from a mixture of *n*-hexane and diethyl ether.

The crude reddish brown solid (39.8 mg), obtained by a similar manner, was purified by preparative HPLC (Cosmosil 5C_18_ MS-II (20 mm × 250 mm, i.d., Nakalai Tesque Inc.), MeOH–0.03% aqueous Et_2_NH (70:30, *v/v*)) to give **2** (21.1 mg, 42%) and **2a** (18.1 mg, 41%).

Compound **2′′**: IR (KBr) *v*_max_ cm^−1^: 2927, 2720–2500, 1721, 1450, 1261, 1033; ^1^H- and ^13^C-NMR spectroscopic data, see Table 6; positive-ion ESIMS *m/z* 282 [M − C_19_H_18_NO_4_]^+^; HREIMS *m/z* 282.1483 (calcd for C_18_H_20_NO_2_ , 282.1489).

Compound **2a**: An amorphous powder; ${\left[\alpha \right]}_{D}^{25}$ −160.9 (*c* 0.19, CHCl_3_); UV [MeOH, nm (log *ε*)]: 229 (4.30), 272 (4.12); IR (KBr) *v*_max_ cm^−1^: 2927, 1685, 1446, 1246, 1107; ^1^H- and ^13^C-NMR spectroscopic data, see Table 6; positive-ion ESIMS *m/z*: 362 [M + Na]^+^; HRESIMS *m/z*: 362.1361 [M + Na]^+^ (calcd for C_20_H_21_NO_4_Na, 362.1363).

### 3.10. Reagents for Bioassays

Dulbecco′s modified Eagle′s medium (DMEM, 4.5 g/L glucose) was purchased from Sigma–Aldrich (St. Louis, MO, USA); fetal bovine serum (FBS), penicillin, and streptomycin were purchased from Gibco (Invitrogen, Carlsbad, CA, USA); and other chemicals used in this study were purchased from Wako Pure Chemical Co., Ltd. (Osaka, Japan). The 48- and 96-well microplates (Sumilon) were purchased from Sumitomo Bakelite Co., Ltd. (Tokyo, Japan).

### 3.11. Cell Culture

Murine B16 melanoma 4A5 cells (RCB0557) were obtained from Riken Cell Bank (Tsukuba, Japan). The cells were grown in DMEM (glucose; 4500 mg/L) supplemented with 10% FBS, penicillin (100 units/mL), and streptomycin (100 µg/mL) at 37 °C in 5% CO_2_/air. The cells were harvested by incubation in phosphate-buffered saline (PBS) containing 0.05% ethylenediaminetetraacetic acid (EDTA) and 0.02% trypsin for ca. 5 min at 37 °C and used for the subsequent bioassays.

### 3.12. Melanogenesis and Cell Viability

Effects on theophylline-stimulated melanogenesis and viability of B16 melanoma 4A5 cells were examined according to the protocol described previously [25,26,27].

### 3.13. Total Contents (%) of the 10 Alkaloids *(**1**–**10**)* Calculated Based on the Ratio of IC_50_ Values (µg/mL) Against Melanogenesis Inhibitory Activities

Total contents (%) of the 10 alkaloids (**1**–**10**) are presented in reduced values to the content of nuciferine (**1**), and were calculated out by the following equation.

Total content (%) = [(content (%) of **1**)] + [(content (%) of **2**) × (IC_50_ of **1**)/(IC_50_ of **2**)] + [(content (%) of **3**) × (IC_50_ of **1**)/(IC_50_ of **3**)] + [(content (%) of **4**) × (IC_50_ of **1**)/(IC_50_ of **4**)] + [(content (%) of **5**) × (IC_50_ of **1**)/(IC_50_ of **5**)] + [(content (%) of **6**) × (IC_50_ of **1**)/(IC_50_ of **6**)] + [(content (%) of **7**) × (IC_50_ of **1**)/(IC_50_ of **7**)] + [(content (%) of **8**) × (IC_50_ of **1**)/(IC_50_ of **8**)] + [(content (%) of **9**) × (IC_50_ of **1**)/(IC_50_ of **9**)] + [(content (%) of **10**) × (IC_50_ of **1**)/(IC_50_ of **10**)].

### 3.14. Mushroom Tyrosinase

Tyrosinase activities using l-tyrosine or 3,4-dihydroxyphenyl-l-alanine (l-DOPA) as a substrate were determined according to the protocol described previously [25,26,27].

### 3.15. Expressions of Tyrosinase, TRP-1, and TRP-2 mRNA

The expressions of tyrosinase, tyrosine-related protein (TRP)-1, and TRP-2 mRNA were assessed according to the previously reported method [25,27].

### 3.16. Statistical Analysis

Values are expressed as mean ± S.E.M. One-way analysis of variance followed by Dunnett′s test was used for statistical analyses. Probability (*p*) values less than 0.05 were considered significant.

## 4. Conclusions

We have developed a practical method for the simultaneous quantitative determination of five aporphine alkaloids, nuciferine (**1**), nornuciferine (**2**), *N*-methylasimilobine (**3**), asimilobine (**4**), and pronuciferine (**5**), and five benzylisoquinoline alkaloids, armepavine (**6**), norarmepavine (**7**), *N*-methylcoclaurine (**8**), coclaurine (**9**), and norjuziphine (**10**), in the lotus flower (the flower buds of *N. nucifera*). The method was validated with respect to linearity, detection limit, precision, and accuracy. The assay was reproducible and precise, and could be readily utilized for evaluation of the theophylline-stimulated melanogenesis inhibitory activity in B16 melanoma 4A5 cells of extracts of the lotus flower and related products. Among the active alkaloids, compound **2** was found to absorb CO_2_ from the air to give a reversible carbamate salt (**2′′**) via formation of unstable carbamic acids (**2′**). To clarify the efficacy of the established quantitative analysis of 10 alkaloids (**1**–**10**) as a quality control for lotus flower, correlations between the total alkaloid contents and the melanogenesis inhibitory activities of the corresponding extracts were examined. As a result, precise and strict correlations between this analytical method and the melanogenesis inhibitory activities were achieved.

## Figures and Tables

**Table 1:** Inhibitory effects of the methanol extract and its fractions from lotus flower on theophylline-stimulated melanogenesis and viability in B16 4A5 cells.

Treatment ^a^	Inhibition (%)					IC_50_ (µg/mL)
	0 µg/mL	3 µg/mL	10 µg/mL	30 µg/mL	100 µg/mL	
MeOH ext.	0.0 ± 1.3 (100.0 ± 9.3)	28.3 ± 3.7 (110.9 ± 2.7)	68.9 ± 3.1 ** (124.2 ± 5.1)	96.4 ± 3.1 ** (126.5 ± 5.3)	97.0 ± 3.3 ** (103.3 ± 7.1)	5.6
EtOAc-soluble fraction	0.0 ± 6.1 (100.0 ± 7.2)	11.0 ± 1.7 (99.3 ± 9.8)	51.5 ± 5.3 ** (106.8 ± 5.1)	83.0 ± 2.9 ** (110.9 ± 4.2)	100.9 ± 3.4 ** (118.3 ± 10.4)	11.1
*n*-BuOH-soluble fraction	0.0 ± 13.3 (100.0 ± 11.2)	17.6 ± 7.0 (104.6 ± 11.6)	35.9 ± 7.1 (104.9 ± 2.6)	72.3 ± 4.7 ** (114.8 ± 13.9)	94.7 ± 3.5 ** (104.3 ± 3.8)	13.7
**Treatment**	**Inhibition (%)**					**IC_50_** **(µg/mL)**
	**0 µg/mL**	**0.1 µg/mL**	**0.3 µg/mL**	**1 µg/mL**	**3 µg/mL**	
CHCl_3_-soluble fraction	0.0 ± 1.7 (100.0 ± 3.5)	19.8 ± 6.8 (101.5 ± 6.1)	35.6 ± 6.6 ** (103.1 ± 6.1)	79.2 ± 2.3 ** (114.7 ± 4.6)	107.2 ± 3.0 ** (107.4 ± 9.5)	0.37

Each value represents the mean ± S.E.M. (*n* = 4); asterisks denote significant differences from the control group, ** *p* < 0.01; ^a^ Bioassay-guided separation study was carried out using the flower buds of *N. nucifera* originating in Thailand (NN-1).

**Table 2:** Extraction efficiently of alkaloids (**1**–**10**) from lotus flower.

Extraction Method	Extraction Yield (%)	Contents (mg/g in Dry Material) ^a^										Total
		1	2	3	4	5	6	7	8	9	10	
Methanol, reflux	15.0	1.76 (100)	1.75 (100)	0.07 (100)	0.63 (100)	0.69 (100)	0.83 (100)	1.45 (100)	5.73 (100)	1.30 (100)	0.75 (100)	14.96 (100)
50% Methanol, reflux	25.3	1.09 (62)	1.35 (77)	0.05 (71)	0.50 (79)	0.61 (88)	0.78 (94)	1.35 (93)	3.79 (66)	0.94 (73)	0.56 (75)	11.02 (74)
H_2_O, reflux	23.1	0.24 (14)	0.35 (20)	n.d. ^b^	0.21 (33)	0.18 (26)	0.38 (45)	0.78 (54)	2.57 (45)	0.66 (51)	0.29 (38)	5.66 (38)
Methanol, sonication	9.6	0.88 (50)	1.11 (64)	0.03 (44)	0.39 (62)	0.33 (48)	0.47 (56)	0.97 (67)	2.77 (48)	0.70 (54)	0.42 (56)	8.07 (54)
50% Methanol, sonication	22.0	0.98 (56)	1.27 (73)	0.04 (58)	0.49 (78)	0.47 (69)	0.80 (96)	1.38 (95)	3.93 (69)	0.97 (75)	0.59 (79)	10.92 (73)
H_2_O, sonication	19.3	0.14 (8)	0.21 (12)	n.d. ^b^	0.12 (20)	0.08 (11)	0.25 (30)	0.53 (37)	1.91 (33)	0.48 (37)	0.19 (26)	3.91 (26)

Extraction efficiently was tested using NN-1 (loss of drying 10.33%); ^a^ relative value (%) against the content obtained by methanol under reflux is given in parentheses; ^b^ less than the quantitation limit.

**Table 3:** Linearities, detection and quantitation limits, and precisions for alkaloids (**1**–**10**) in lotus flower.

Analyte	Regression Equation ^a^	Correlation Coefficient	Detection Limit ^b^ (ng)	Quantitation Limit ^b^ (ng)	Precision ^c^ (RSD, %)	
					Intra-Day	Inter-Day
Nuciferine (**1**)	*y* = 7477635*x* − 1302	0.9998	0.17	0.51	0.25	0.59
Nornuciferine (**2**)	*y* = 2698708*x* − 10941	1.0000	0.71	2.16	0.79	0.43
*N*-Methylasimilobine (**3**)	*y* = 7054297*x* + 243961	0.9996	0.32	0.99	1.36	1.40
Asimilobine (**4**)	*y* = 2076494*x* − 36021	0.9999	0.70	2.13	0.63	0.57
Pronuciferine (**5**)	*y* = 3522995*x* + 101328	0.9998	0.73	2.18	0.95	1.08
Armepavine (**6**)	*y* = 2076494*x* − 36021	0.9999	0.32	0.97	0.68	1.10
Norarmepavine (**7**)	*y* = 1998354*x* − 15296	0.9999	0.81	2.47	0.54	0.73
*N*-Methylcoclaurine (**8**)	*y* = 1595194*x* + 53314	0.9999	0.90	2.71	0.59	0.86
Coclaurine (**9**)	*y* = 1878370*x* + 16838	0.9999	0.44	1.33	0.98	0.39
Norjuziphine (**10**)	*y* = 1745634*x* + 15240	1.0000	0.88	2.65	0.64	0.66

^a^ In the regression equation, *x* is the concentration of the analyte solution (µg/mL), and *y* is the peak area of the analyte; ^b^ values are the amount of the analyte injected on-column and ^c^ precision of the analytical method were tested using the methanol extract of NN-1 (*n* = 5).

**Table 4:** Recoveries for alkaloids (**1**–**10**) from lotus flower.

Add (µg/mL)	Recovery ^a^ (%)									
	1	2	3	4	5	6	7	8	9	10
10	98.7 ± 0.6	101.4 ± 0.7	95.3 ± 1.0	99.0 ± 0.9	93.2 ± 0.7	98.5 ± 0.3	104.0 ± 0.7	94.7 ± 1.4	97.4 ± 0.2	97.4 ± 1.0
15	92.3 ± 0.4	101.7 ± 0.2	97.5 ± 0.7	101.8 ± 0.1	98.1 ± 0.5	102.2 ± 0.6	99.4 ± 1.1	99.8 ± 1.6	104.0 ± 0.2	100.2 ± 0.8
20	98.4 ± 0.1	105.8 ± 0.7	101.2 ± 0.8	102.0 ± 0.7	95.9 ± 0.8	105.1 ± 0.6	99.9 ± 0.9	96.3 ± 1.1	105.8 ± 0.3	95.3 ± 0.8

^a^ The recovery rates were determined by adding analytes of three different concentrations (10, 15, and 20 µg/mL) to the sample solution; recoveries spiked with the methanol extract of NN-1 (each 400 µg/mL, *n* = 3).

**Table 5:** Contents of alkaloids (**1**–**10**) in the methanol extracts from lotus flower.

Sample No.	Part	Loss of Drying ^a^ (%)	Extraction Yield ^b^ (%)	Contents (mg/g in Dry Material) ^a^										Total
				1	2	3	4	5	6	7	8	9	10	
NN-1	whole flowers	10.3	15.0	1.76	1.75	0.07	0.63	0.69	0.83	1.45	5.73	1.30	0.75	14.96
NN-2	petals	8.6	16.6	1.99	2.41	0.07	1.22	0.62	1.02	1.74	5.85	1.60	0.52	17.04
NN-3	receptacles	10.0	8.6	0.06	0.07	n.d. ^c^	0.01	0.01	0.01	0.01	0.20	0.04	n.d. ^c^	0.41
NN-4	stamens	8.6	20.0	0.58	0.68	n.d. ^c^	0.25	0.23	0.47	0.64	2.88	0.60	0.32	6.65
NN-5	whole flowers	8.1	16.3	0.56	0.27	n.d. ^c^	0.04	n.d. ^c^	0.23	0.34	1.74	0.19	0.16	3.53
NN-6	petals	7.3	19.0	0.80	0.34	n.d. ^c^	n.d. ^c^	0.01	0.36	0.52	3.14	0.32	0.27	5.76
NN-7	receptacles	9.2	7.4	0.27	0.53	n.d. ^c^	n.d. ^c^	0.05	0.03	0.03	0.74	0.27	n.d.^c^	1.92
NN-8	stamens	7.9	15.5	0.01	n.d. ^c^	n.d. ^c^	n.d. ^c^	n.d. ^c^	n.d. ^c^	n.d. ^c^	0.03	0.01	0.03	0.08

^a^ Each powdered sample was dried at 105 °C for 8 h; ^b^ each powdered sample was extracted two times with methanol under reflux for 120 min and ^c^ less than the quantitation limit.

**Table 6:** ^1^H- (800 MHz) and ^13^C- (200 MHz) NMR data for **2′′**, **2a**, and original alkaloid **2** in CDCl_3_.

Position	2′′ (anion part)		Position	2′′ (cation part)	
	δ_H_ (*J* in Hz)	δ_C_		δ_H_ (*J* in Hz)	δ_C_
1		146.0 ^a^	1′		146.2 ^a^
2		152.5 ^b^	2′		153.7 ^b^
3	6.69 (s)	111.4	3′	6.67 (s)	111.4
3a		128.7	3a′		125.7
4	2.74 (br d, ca. 15) 2.98 (br d, ca. 15)	29.9, 30.2	4′	2.95 (dd, 3.8, 16.8) 3.62 (ddd, 5.7, 13.5, 16.8)	25.5
5	3.20–3.35 (m) 4.62 (br d, ca. 12.5)	41.8, 44.4	5′	3.24 (br ddd-like, ca. 13.5, 13.5, 13.5) 3.87 (br dd-like, ca. 5.7, 13.5)	41.4
6a	4.92 (br d, ca. 13)	54.9, 55.8	6a′	4.29 (br dd-like, ca. 13.5, 13.5)	53.0
7	2.86–2.90 (m) 3.02–3.14 (m)	33.9, 35.9	7′	3.38 (dd, 13.5, 13.5) 3.45 (dd, 4.5, 13.5)	34.0
7a		135.7	7a′		133.1
8	7.24–7.30 (m)	128.0 ^c^	8′	7.24–7.30 (m)	128.3 ^c^
9	7.24–7.30 (m)	127.4 ^c^	9′	7.24–7.30 (m)	128.2 ^c^
10	7.35 (m)	127.3 ^c^	10′	7.35 (m)	127.8 ^c^
11	8.44 (br s-like)	128.5 ^c^	11′	8.41 (d, 7.9)	128.6 ^c^
11a		131.3	11a′		131.3
11b		127.5 ^d^	11b′		127.0 ^d^
11c		124.8, 124.9	11c′		121.4
1-O*CH*_3_	3.667 ^e^ (s)	60.3 ^f^	1′-O*CH*_3_	3.673 ^e^ (s)	60.0 ^f^
2-O*CH*_3_	3.90 ^g^ (s)	56.0 ^h^	2′-O*CH*_3_	3.91 ^g^ (s)	55.9 ^h^
*N*-*C*OO		157.5, 160.1	*N*H_2_	9.96 (br ddd-like, ca. 13.5, 13.5, 13.5 ) 10.84 (br d-like, ca. 13.5)	
**Position**	**2a**		**Position**	**2**	
	**δ_H_ (*J* in Hz)**	**δ_C_**		**δ_H_ (*J* in Hz)**	**δ_C_**
1		145.7	1		145.3
2		152.1	2		152.2
3	6.67 (s)	111.5	3	6.65 (s)	111.8
3a		126.1	3a		128.5
4	2.65 (br d, ca. 15) 2.88 (m)	30.3	4	2.71 (d, 13.1) 3.05 (m)	28.9
5	3.00 (br dd, ca. 11, 13) 4.73 (br d, ca. 13)	38.8	5	3.01 (m) 3.40 (br q, ca. 6)	43.0
6a	4.46 (br s)	51.4	6a	3.85 (br dd, ca. 5, 14)	53.5
7	2.86 (m) 2.98 (dd, 12.8, 15.8)	35.2	7	2.77 (t, 13.8) 2.87 (dd, 4.6, 13.8)	37.3
7a		136.8	7a		135.9
8	7.25 (dd, 1.6, 7.8)	128.2	8	7.24 (m)	127.8
9	7.27 (br dd, ca. 8, 8)	127.5	9	7.21 (ddd, 1.1, 7.1, 7.1)	127.4
10	7.32 (ddd, 1.6, 7.8, 8.0)	126.9	10	7.30 (m)	127.0
11	8.44 (br d, ca. 8)	128.3	11	8.39 (br d, ca. 8)	128.4
11a		131.7	11a		132.1
11b		127.6	11b		126.5
11c		129.7	11c		128.7
1-O*CH*_3_	3.66 (s)	60.2	1-O*CH*_3_	3.67 (s)	60.2
2-O*CH*_3_	3.90 (s)	56.0	2-O*CH*_3_	3.88 (s)	55.9
*N*-*C*OO		156.0			
*N*-CO_2_*CH*_3_	3.76 (s)	52.6			

^a–h^ May be interchangeable.

**Table 7:** Inhibitory effects of the alkaloids (**1**–**10**) and **2a** on theophylline-stimulated melanogenesis and viability in B16 4A5 cells.

Treatment	Inhibition (%)					IC_50_	
	0 µM	3 µM	10 µM	30 µM	100 µM	(µM)	(µg/mL) ^b^
Nuciferine·HCl (**1**) ^a^	0.0 ± 4.0 (100.0 ± 2.6)	28.3 ± 4.2 ** (112.1 ± 2.6)	57.8 ± 2.3 ** (112.1 ± 2.5)	89.7 ± 1.7 ** (100.9 ± 5.8)	91.8 ± 2.6 ** (45.9 ± 3.3 ^#^)	7.1	2.4
Nornuciferine·HCl (**2**) ^a^	0.0 ± 3.4 (100.0 ± 4.9)	44.6 ± 3.4 ** (99.5 ± 8.2)	70.6 ± 3.6 ** (93.9 ± 4.1)	94.3 ± 3.2 ** (70.6 ± 4.8 ^#^)	— (1.9 ± 0.1 ^#^)	3.9	1.2
*N*-Methylasimilobine·HCl (**3**) ^a^	0.0 ± 4.7 (100.0 ± 3.9)	3.3 ± 2.9 (96.6 ± 2.6)	19.5 ± 2.4 ** (100.4 ± 3.0)	63.4 ± 5.2 ** (119.3 ± 9.4)	89.8 ± 2.1 ** (57.7 ± 1.3 ^#^)	43.1	13.7
Asimilobine·HCl (**4**) ^a^	0.0 ± 8.1 (100.0 ± 5.7)	16.2 ± 12.8 (96.5 ± 7.5)	31.9 ± 2.2 ** (98.9 ± 10.0)	87.2 ± 3.3 ** (76.6 ± 6.3)	— (13.7 ± 2.2 ^#^)	11.3	3.4
Pronuciferine·HCl (**5**) ^a^	0.0 ± 10.7 (100.0 ± 4.5)	23.1 ± 3.4 (104.2 ± 3.4)	18.8 ± 1.2 (97.4 ± 0.7)	37.2 ± 2.6 ** (98.4 ± 1.8)	88.4 ± 3.7 ** (89.1 ± 9.4)	47.1	16.3
Armepavine·HCl (**6**) ^a^	0.0 ± 6.0 (100.0 ± 2.5)	33.9 ± 0.8 ** (104.0 ± 3.3)	58.5 ± 7.1 ** (104.0 ± 7.7)	81.8 ± 3.0 ** (104.0 ± 7.2)	97.4 ± 0.4 ** (78.1 ± 2.0)	6.5	3.4
Norarmepavine·HCl (**7**) ^a^	0.0 ± 3.1 (100.0 ± 2.6)	32.6 ± 4.4 ** (86.4 ± 4.0)	53.5 ± 8.5 ** (81.2 ± 4.0)	81.6 ± 1.4 ** (83.3 ± 2.2)	90.5 ± 1.2 ** (68.8 ± 1.4 ^#^)	7.5	2.5
*N*-Methylcoclaurine·HCl (**8**) ^a^	0.0 ± 5.4 (100.0 ± 3.0)	38.6 ± 2.4 ** (97.1 ± 1.5)	55.7 ± 3.4 ** (92.8 ± 4.1)	74.7 ± 2.0 ** (96.4 ± 4.2)	— —	6.5	2.2
Coclaurine·HCl (**9**) ^a^	0.0 ± 2.9 (100.0 ± 2.9)	45.6 ± 7.7 ** (97.0 ± 7.7)	65.4 ± 2.5 ** (96.2 ± 5.0)	82.4 ± 3.5 ** (86.5 ± 7.7)	68.0 ± 6.4 ** (53.1 ± 8.3 ^#^)	3.9	1.3
Norjuziphine·HCl (**10**) ^a^	0.0 ± 5.4 (100.0 ± 5.3)	18.5 ± 4.0 * (98.9 ± 6.0)	36.8 ± 4.5 ** (91.3 ± 4.9)	94.4 ± 2.0 ** (83.9 ± 8.3)	106.0 ± 2.0 ** (57.0 ± 2.4 ^#^)	14.4	4.6
**2a**	0.0 ± 7.3 (100.0 ± 4.7)	13.1 ± 8.9 (109.8 ± 4.3)	43.1 ± 7.8 ** (127.5 ± 4.8)	54.5 ± 4.3 ** (129.1 ± 2.8)	— —	19.9	6.7
**Treatment**	**Inhibition (%)**					**IC_50_**	
	**0 µM**	**30 µM**	**100 µM**	**300 µM**	**1000 µM**	**(µM)**	**(µg/mL)**
Arbutin [25,26,27]	0.0 ± 1.4 (100.0 ± 2.1)	20.4 ± 0.5 (82.4 ± 3.0)	38.1 ± 0.9 ** (78.1 ± 1.9)	61.5 ± 0.6 ** (79.8 ± 2.2)	83.7 ± 0.5 ** (53.1 ± 1.8 ^#^)	174	47.4

Each value represents the mean ± S.E.M. (*n* = 4); asterisks denote significant differences from the control group, * *p* < 0.05, ** *p* < 0.01; ^#^ cytotoxic effects were observed, and values in parentheses indicate cell viability (%) in MTT assay; commercial arubutin was purchased from Nakalai Tesque Inc., (Kyoto, Japan); ^a^ each alkaloid was evaluated by its hydrochloride salt; ^b^ each IC_50_ value was converted to µg/mL of corresponding free alkaloid.

**Table 8:** Effects on activity of tyrosinase from mushroom.

Substrate:Treatment	Inhibition (%)					
	l-Tyrosine			l-DOPA		
	0 µM	10 µM	100 µM	0 µM	10 µM	100 µM
Nuciferine·HCl (**1**) ^a^	0.0 ± 3.2	6.5 ± 3.3	30.4 ± 1.9 **	0.0 ± 2.9	4.4 ± 3.8	11.0 ± 3.4
Nornuciferine·HCl (**2**) ^a^	0.0 ± 2.6	1.0 ± 0.7	14.2 ± 1.5 **	0.0 ± 3.1	15.4 ± 4.1	8.7 ± 4.1
*N*-Methylasimilobine·HCl (**3**) ^a^	0.0 ± 3.6	7.2 ± 7.3	0.8 ± 3.5	0.0 ± 1.5	2.3 ± 1.5	3.1 ± 3.9
Asimilobine·HCl (**4**) ^a^	0.0 ± 1.8	10.5 ± 2.5 *	14.0 ± 1.2 **	0.0 ± 3.3	3.2 ± 2.4	5.7 ± 3.0
Pronuciferine·HCl (**5**) ^a^	0.0 ± 5.3	−0.4 ± 2.4	−4.4 ± 8.1	0.0 ± 2.0	6.5 ± 2.0	5.3 ± 5.2
Armepavine·HCl (**6**) ^a^	0.0 ± 4.4	−1.9 ± 0.9	40.2 ± 3.7 **	0.0 ± 0.5	4.5 ± 0.8	2.9 ± 1.2
Norarmepavine·HCl (**7**) ^a^	0.0 ± 1.8	−1.7 ± 1.5	23.3 ± 1.6 **	0.0 ± 2.7	−1.3 ± 1.4	1.5 ± 1.5
*N*-Methylcoclaurine·HCl (**8**) ^a^	0.0 ± 5.2	−5.0 ± 5.7	15.3 ± 4.3	0.0 ± 2.3	6.1 ± 1.4	7.4 ± 0.8
Coclaurine·HCl (**9**) ^a^	0.0 ± 2.3	9.0 ± 0.7 *	35.1 ± 1.7 **	0.0 ± 2.5	−4.9 ± 1.1	4.4 ± 1.1
Norjuziphine·HCl (**10**) ^a^	0.0 ± 2.5	5.1 ± 1.1	27.4 ± 3.0 **	0.0 ± 2.0	1.8 ± 0.7	22.3 ± 3.6 **
**2a**	0.0 ± 1.5	5.3 ± 1.7	2.9 ± 0.7	0.0 ± 2.7	3.2 ± 1.1	6.6 ± 2.3
**Substrate: l-Tyrosine**	**Inhibition (%)**					
**Treatment**	**0 µM**	**10 µM**	**30 µM**	**100 µM**	**300 µM**	**IC_50_ (µM)**
Kojic acid [25,26,27]	0.0 ± 2.4	12.2 ± 3.3	46.4 ± 2.6 **	66.5 ± 2.1 **	96.8 ± 0.9 **	43.6
**Substrate: l-DOPA**	**Inhibition (%)**					
**Treatment**	**0 µM**	**10 µM**	**30 µM**	**100 µM**	**300 µM**	**IC_50_ (µM)**
Kojic acid [25,26,27]	0.0 ± 0.9	22.3 ± 2.1 **	50.6 ± 0.6 **	78.2 ± 0.7 **	89.3 ± 0.3 **	29.6

Each value represents the mean ± S.E.M. (*n* = 4); asterisks denote significant differences from the control group, * *p* < 0.05, ** *p* < 0.01; commercial kojic acid was purchased from Nakalai Tesque Inc., (Kyoto, Japan); ^a^ each alkaloid was evaluated by its hydrochloride salt.

**Table 9:** Effects of **1**, **2**, **6**, **7**, and **9** on expression of tyrosinase, TRP-1, and TRP-2 mRNA in B16 4A5 cells.

Treatment	Tyrosinase mRNA/-actin mRNA		
	0 µM	3 µM	10 µM
Nuciferine·HCl (**1**) ^a^	1.00 ± 0.15	0.59 ± 0.03 *	0.45 ± 0.05 *
Nornuciferine·HCl (**2**) ^a^	1.00 ± 0.19	0.76 ± 0.05	0.51 ± 0.10
Armepavine·HCl (**6**) ^a^	1.00 ± 0.14	0.86 ± 0.13	0.74 ± 0.02
Norarmepavine·HCl (**7**) ^a^	1.00 ± 0.24	0.81 ± 0.08	1.00 ± 0.11
Coclaurine·HCl (**9**) ^a^	1.00 ± 0.14	0.82 ± 0.21	0.52 ± 0.05
**Treatment**	**TRP-1 mRNA/-actin mRNA**		
	**0 µM**	**3 µM**	**10 µM**
Nuciferine·HCl (**1**) ^a^	1.00 ± 0.12	1.18 ± 0.17	1.18 ± 0.25
Nornuciferine·HCl (**2**) ^a^	1.00 ± 0.10	1.21 ± 0.18	1.15 ± 0.19
Armepavine·HCl (**6**) ^a^	1.00 ± 0.22	0.98 ± 0.32	0.83 ± 0.15
Norarmepavine·HCl (**7**) ^a^	1.00 ± 0.09	0.87 ± 0.22	1.03 ± 0.25
Coclaurine·HCl (**9**) ^a^	1.00 ± 0.24	0.51 ± 0.08	0.66 ± 0.13
**Treatment**	**TRP-2 mRNA/-actin mRNA**		
	**0 µM**	**3 µM**	**10 µM**
Nuciferine·HCl (**1**) ^a^	1.00 ± 0.16	1.33 ± 0.35	0.87 ± 0.16
Nornuciferine·HCl (**2**) ^a^	1.00 ± 0.12	0.92 ± 0.20	1.36 ± 0.09
Armepavine·HCl (**6**) ^a^	1.00 ± 0.05	1.07 ± 0.22	0.81 ± 0.22
Norarmepavine·HCl (**7**) ^a^	1.00 ± 0.18	0.80 ± 0.24	0.77 ± 0.14
Coclaurine·HCl (**9**) ^a^	1.00 ± 0.11	1.02 ± 0.22	1.07 ± 0.07

Each value represents the mean ± S.E.M. (*n* = 3); asterisks denote significant differences from the control group, * *p* < 0.05; ^a^ each alkaloid was evaluated by its hydrochloride salt.

**Table 10:** Inhibitory effects of the methanol extract from lotus flower (NN-1–NN-8) on theophylline-stimulated melanogenesis and viability in B16 4A5 cells.

Sample No.	Inhibition (%)					IC_50_
	0 µg/mL	3 µg/mL	10 µg/mL	30 µg/mL	100 µg/mL	(µg/mL)
NN-1	0.0 ± 1.3 (100.0 ± 9.3)	28.3 ± 3.7 (110.9 ± 2.7)	68.9 ± 3.1 ** (124.2 ± 5.1)	96.4 ± 3.1 ** (126.5 ± 5.3)	97.0 ± 3.3 ** (103.3 ± 7.1)	5.6
NN-2	0.0 ± 5.7 (100.0 ± 2.7)	15.7 ± 8.2 (113.3 ± 2.2)	49.9 ± 12.0 ** (114.6 ± 1.8)	108.2 ± 4.1 ** (128.9 ± 2.8)	103.8 ± 6.7 ** (116.2 ± 2.5)	8.8
NN-3	0.0 ± 5.9 (100.0 ± 6.8)	18.6 ± 4.2 (104.7 ± 4.3)	26.1 ± 5.0 ** (102.6 ± 2.9)	72.9 ± 0.7 ** (119.3 ± 5.4)	86.8 ± 4.1 ** (108.2 ± 3.6)	34.7
NN-4	0.0 ± 10.0 (100.0 ± 5.3)	10.7 ± 8.1 (99.4 ± 7.1)	28.3 ± 4.7 (99.1 ± 11.1)	95.7 ± 1.6 ** (98.0 ± 5.0)	107.0 ± 2.1 ** (92.4 ± 6.3)	24.0
NN-5	0.0 ± 7.3 (100.0 ± 3.7)	23.7 ± 4.0 ** (99.6 ± 2.2)	34.4 ± 5.1 ** (102.7 ± 4.3)	86.6 ± 2.6 ** (99.6 ± 2.4)	103.4 ± 1.9 ** (92.5 ± 5.0)	19.9
NN-6	0.0 ± 6.0 (100.0 ± 4.5)	−6.9 ± 13.7 (87.8 ± 4.7)	−6.3 ± 12.0 (88.1 ± 1.0)	77.4 ± 5.4 ** (90.8 ± 4.0)	104.6 ± 2.1 ** (74.1 ± 3.3 ^#^)	54.1
NN-7	0.0 ± 4.4 (100.0 ± 1.6)	−9.5 ± 6.9 (94.7 ± 4.1)	9.0 ± 7.6 (101.2 ± 2.2)	79.4 ± 2.1 ** (102.3 ± 2.8)	84.2 ± 4.2 ** (84.9 ± 0.9)	46.6
NN-8	0.0 ± 1.7 (100.0 ± 4.6)	−3.3 ± 4.8 (100.8 ± 7.4)	1.3 ± 7.9 (93.5 ± 3.2)	57.6 ± 3.1 ** (95.6 ± 3.2)	64.9 ± 2.3 ** (97.8 ± 3.8)	78.9

Each value represents the mean ± S.E.M. (*n* = 4); asterisks denote significant differences from the control group, ** *p* < 0.01.; ^#^ cytotoxic effects were observed, and values in parentheses indicate cell viability (%) in MTT assay.

## Supplementary Materials

Supplementary materials can be accessed at: http://www.mdpi.com/1420-3049/21/7/930/s1.
