# Supplementary material for: Quantitative Determination of Alkaloids in Lotus Flower (Flower Buds of Nelumbo nucifera) and Their Melanogenesis Inhibitory Activity
Source: Molecules. 2016 Jul 19;21(7):930. doi: 10.3390/molecules21070930 (PMC6272935; doi:10.3390/molecules21070930)
Supplement: Supplementary file 1 [file molecules-21-00930-s001.pdf]

Supplementary Materials: Quantitative Determination of Alkaloids in Lotus Flower (Flower Buds of *Nelumbo nucifera*) and Their Melanogenesis Inhibitory Activity

Toshio Morikawa, Niichiro Kitagawa, Genzoh Tanabe, Kiyofumi Ninomiya, Shuhei Okugawa, Chiaki Motai, Iyori Kamei, Masayuki Yoshikawa, I-Jung Lee [5] and Osamu Muraoka

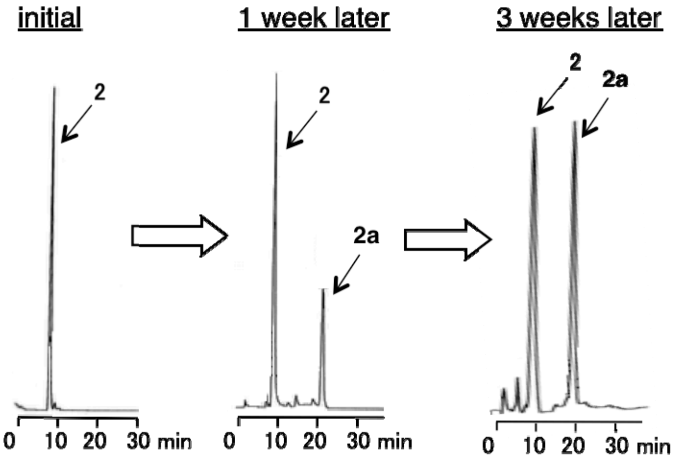

Figure S1. Monitoring for chemical transformation of **2** into its ammonium carbamate salt (**2''**) and to methyl carbamate (**2a**) by HPLC analysis.

Table S1. Contents of alkaloids (**1–10**) in the methanol extracts from the leaf, fruit, and embryo parts of *N. nucifera*.

Sample No.	Part	Loss of Drying [a] (%)	Extraction Yield [b] (%)	Contents (mg/g in Dry Material)										Total [a]
				1	2	3	4	5	6	7	8	9	10	
NN-9	Leaf	9.8	19.0	0.24	0.05	0.38	0.14	n.d. [c]	0.16	0.25	0.16	n.d. [c]	0.07	1.20
NN-10	Fruit	10.9	10.9	n.d. [c]	n.d. [c]	n.d. [c]	n.d. [c]	n.d. [c]	n.d. [c]	n.d. [c]	n.d. [c]	n.d. [c]	n.d. [c]	n.d. [c]
NN-11	Fruit	9.3	10.0	n.d. [c]	n.d. [c]	n.d. [c]	n.d. [c]	n.d. [c]	n.d. [c]	n.d. [c]	n.d. [c]	n.d. [c]	n.d. [c]	n.d. [c]
NN-12	Embryo	8.4	32.8	0.02	n.d. [c]	n.d. [c]	n.d. [c]	n.d. [c]	0.25	n.d. [c]	0.20	n.d. [c]	0.17	0.64

[a] Each powdered sample was dried at 105°C for 8 h; [b] each powdered sample was extracted two times with methanol under reflux for 120 min and [c] less than the quantitation limit.

Table S2. Contents of alkaloids (**1**–**10**) in the methanol extracts from the leaf (NN-9), fruit (NN-10 and 11), and embryo (NN-12) of *N. nucifera*.

Sample No.	Inhibition (%)					IC50 (μg/mL)
	0 μg/mL	3 μg/mL	10 μg/mL	30 μg/mL	100 μg/mL	
NN-9	0.0 ± 4.9<br>(100.0 ± 3.4)	15.0 ± 5.7<br>(66.2 ± 2.6 #)	7.6 ± 6.3<br>(58.8 ± 1.9 #)	15.6 ± 8.0<br>(51.7 ± 3.1 #)	—<br>(39.5 ± 1.7 #)	>100
NN-10	0.0 ± 3.6<br>(100.0 ± 4.9)	22.7 ± 9.2<br>(112.2 ± 3.2)	8.4 ± 4.8<br>(103.9 ± 4.9)	17.5 ± 5.6<br>(119.7 ± 3.8)	11.6 ± 1.5<br>(126.8 ± 4.9)	>100
NN-11	0.0 ± 7.6<br>(100.0 ± 2.1)	11.9 ± 6.8<br>(99.3 ± 2.8)	2.8 ± 4.1<br>(92.2 ± 5.9)	22.8 ± 4.9<br>(102.8 ± 2.2)	42.8 ± 4.8 **<br>(113.2 ± 6.9)	>100
NN-12	0.0 ± 10.9<br>(100.0 ± 5.9)	30.8 ± 15.4<br>(107.8 ± 5.0)	88.0 ± 5.0 **<br>(95.5 ± 5.8)	92.9 ± 2.8 **<br>(84.8 ± 4.5)	71.6 ± 1.9 **<br>(56.2 ± 2.7 #)	4.5

Each value represents the mean ± S.E.M. ($n = 4$); asterisks denote significant differences from the control group, ** $p < 0.01$.; #cytotoxic effects were observed, and values in parentheses indicate cell viability (%) in MTT assay.
